# Cine-cardiac magnetic resonance to distinguish between ischemic and non-ischemic cardiomyopathies: a machine learning approach

**DOI:** 10.1007/s00330-024-10640-8

**Published:** 2024-03-07

**Authors:** Riccardo Cau, Francesco Pisu, Alessandra Pintus, Vitanio Palmisano, Roberta Montisci, Jasjit S. Suri, Rodrigo Salgado, Luca Saba

**Affiliations:** 1 Department of Radiology, Azienda Ospedaliero Universitaria (A.O.U.), di Cagliari – Polo di Monserrato s.s. 554 Monserrato, 09045 Cagliari, Italy; 2 Ospedale General Regionale F. Miulli, Acquaviva Delle Fonti, Italy; 3 Department of Cardiology, Azienda Ospedaliero Universitaria (A.O.U.), di Cagliari – Polo di Monserrato s.s. 554 Monserrato, 09045 Cagliari, Italy; 4 Stroke Monitoring and Diagnostic Division, AtheroPoint™, Roseville, CA USA; 5 Universitair Ziekenhuis Antwerpen, Edegem, Belgium

**Keywords:** Cine magnetic resonance imaging, Artificial intelligence, Machine learning, Cardiomyopathy, Cardiovascular diseases

## Abstract

**Objective:**

This work aimed to derive a machine learning (ML) model for the differentiation between ischemic cardiomyopathy (ICM) and non-ischemic cardiomyopathy (NICM) on non-contrast cardiovascular magnetic resonance (CMR).

**Methods:**

This retrospective study evaluated CMR scans of 107 consecutive patients (49 ICM, 58 NICM), including atrial and ventricular strain parameters. We used these data to compare an explainable tree-based gradient boosting additive model with four traditional ML models for the differentiation of ICM and NICM. The models were trained and internally validated with repeated cross-validation according to discrimination and calibration. Furthermore, we examined important variables for distinguishing between ICM and NICM.

**Results:**

A total of 107 patients and 38 variables were available for the analysis. Of those, 49 were ICM (34 males, mean age 60 ± 9 years) and 58 patients were NICM (38 males, mean age 56 ± 19 years). After 10 repetitions of the tenfold cross-validation, the proposed model achieved the highest area under curve (0.82, 95% CI [0.47–1.00]) and lowest Brier score (0.19, 95% CI [0.13–0.27]), showing competitive diagnostic accuracy and calibration. At the Youden’s index, sensitivity was 0.72 (95% CI [0.68–0.76]), the highest of all. Analysis of predictions revealed that both atrial and ventricular strain CMR parameters were important for the identification of ICM patients.

**Conclusion:**

The current study demonstrated that using a ML model, multi chamber myocardial strain, and function on non-contrast CMR parameters enables the discrimination between ICM and NICM with competitive diagnostic accuracy.

**Clinical relevance statement:**

A machine learning model based on non-contrast cardiovascular magnetic resonance parameters may discriminate between ischemic and non-ischemic cardiomyopathy enabling wider access to cardiovascular magnetic resonance examinations with lower costs and faster imaging acquisition.

**Key Points:**

*• The exponential growth in cardiovascular magnetic resonance examinations may require faster and more cost-effective protocols.*

*• Artificial intelligence models can be utilized to distinguish between ischemic and non-ischemic etiologies.*

*• Machine learning using non-contrast CMR parameters can effectively distinguish between ischemic and non-ischemic cardiomyopathies.*

**Graphical Abstract:**

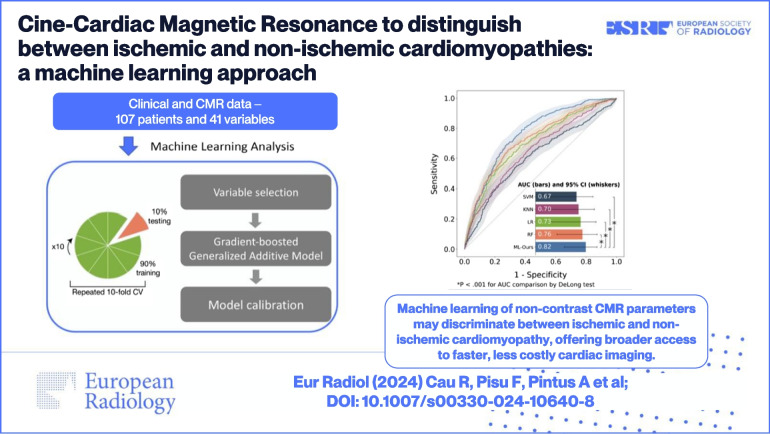

**Supplementary Information:**

The online version contains supplementary material available at 10.1007/s00330-024-10640-8.

## Introduction

Cardiovascular diseases (CVDs) are a major contributor to premature death, with a rising trend due to population growth and aging [1, 2]. Among CVDs, ischemic cardiomyopathy (ICM) is the most prevalent [2], while non-ischemic cardiomyopathy (NICM) represents a heterogeneous group of diseases that can also lead to heart failure, arrhythmias, and death [3]. Distinguishing ICM from NICM is important prognostically and therapeutically but has generally depended on invasive diagnostic techniques such as selective coronary arteriography.

Cardiovascular magnetic resonance (CMR) as reflected in the ESC guidelines [4–7] is a well-validated tool to detect myocardial fibrosis and scar allowing to detection of the specific pattern of late gadolinium enhancement (LGE) corresponding to ICM and different NICM [8].

Due to the exponential growth in CMR examinations, the wider availability of sustainable, faster, and more cost-effective CMR protocol is expected to unquestionably yield significant advantages in real-life clinical practice. In addition, cardiac symptoms such as orthopnea may limit patient tolerability of CMR examinations, and some patients may not be eligible for contrast media administration due to concomitant renal disease.

In recent times, the field of non-contrast CMR examination has seen a significant emergence facilitated by artificial intelligence (AI) models, yielding promising results [9]. A subset of AI, namely machine learning (ML), may overcome the necessity of contrast media administration, expanding the clinical applicability of CMR. The ML-based models can employ hand-crafted features from non-contrast cine-CMR to discriminate ICM from NICM.

In particular, ML-based models have already shown utility in assessing myocardial scar location and extension [10–12], distinguishing chronic from subacute ICM [13], and characterizing different patterns of cardiomyopathy [14, 15]. Previous AI-based cine-CMR research was mainly focused on the application of using radiomics analysis which had its intrinsic limitations, namely being time-consuming, less reproducible, lacking standardization, and prone to mistakes in interpreting the results [16]. To overcome these limitations, the current study investigated well-validated cardiac volumes and functions as well as atrial and ventricular strain analysis.

This work aimed to derive a ML model for the differentiation of ischemic and non-ischemic cardiomyopathies on cine-CMR without contrast.

## Material and method

### Study population

The current study was approved by the Institutional Review Board and informed consent was waived owing to the retrospective nature of the study.

This work involved 240 consecutive patients with reduced left ventricle (LV) ejection fraction who underwent CMR examinations at our institution for viability evaluation and the evaluation of cardiomyopathy etiology between March 3, 2017, and August 7, 2021.

The diagnosis of ischemic etiologies was based on the presence of significant coronary artery disease with more than 50% stenosis on coronary angiography and/or history of previous myocardial infarction or revascularization. The diagnosis of NICM was based on the presence of LV dysfunction (LV ejection fraction < 50%) in the absence of prior myocardial infarction or obstructive stenoses on coronary arteriography.

CMR examinations that were incomplete or not evaluable due to motion or arrhythmia artifacts (*n* = 40) were excluded as well as patients with congenital heart disease (*n* = 25) and structural heart disease (*n* = 40). In addition, 28 cases were also excluded due to inadequate strain analysis, resulting from issues such as poor angle plane, ventricular wall not being fully visible, or low image quality in cine-CMR images.

**Fig. 1:**
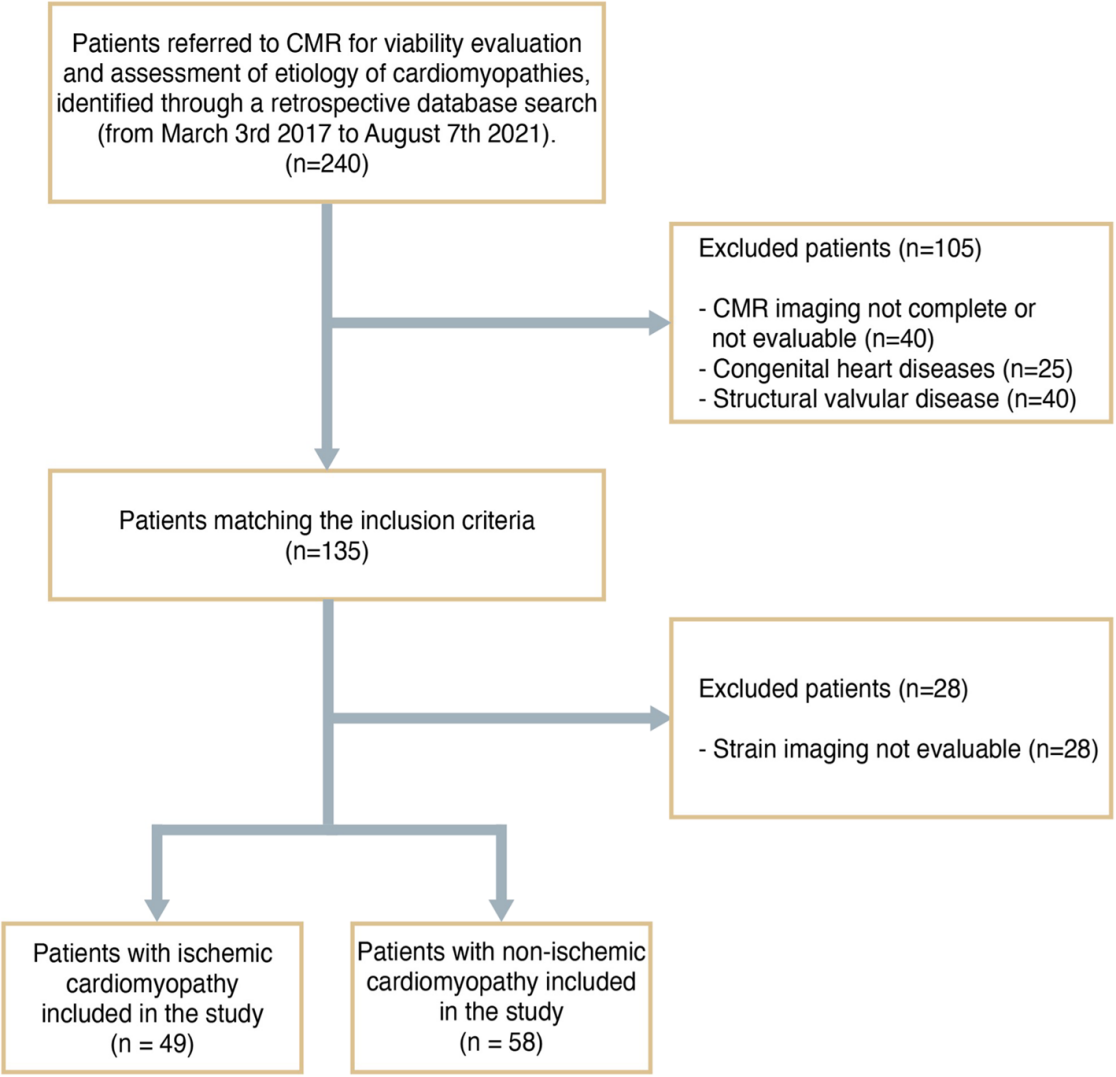
Patient flowchart. Schematic of inclusion and exclusion criteria for this study

After applying inclusion and exclusion criteria, 107 patients including 49 ICM and 58 NICM were included. A flowchart demonstrating the application of inclusion and exclusion criteria is provided in Fig. 1.

### CMR acquisition

CMR scans were performed at 4.1 ± 2.6 days (median = 1 day, range = 1–10 days) after admission to the hospital by using a Philips Achieva dStream 1.5-T scanner system (*Philips Healthcare*). Anterior coil arrays were used. All cine-images were acquired using a balanced steady-state free precession and retrospective gating during an expiratory breath-hold manoeuvres (TE 1.7 ms; TR 3.4 ms/flip angle 45°, section thickness = 8 mm) in both long-axis (two-, three-, and four-chamber view) as well as short-axis plane with whole ventricular coverage from base to apex.

### CMR image post-processing

We used the commercially available software Circle CVI42 (CVI42, Circle Cardiovascular Imaging Inc.) for cardiac MRI feature tracking (CMR-FT) data analysis. Offline CMR-FT analyses were conducted for evaluation of peak global longitudinal strain (LS), global radial strain (RS), and global circumferential strain (CS) in a 16-segment software-generated 2D model. On all images, the epi- and endocardial borders were traced in end-diastole. After that, an automatic computation was triggered, by which the applied software algorithm automatically outlined the border throughout the cardiac cycle.

LA endocardial borders were manually traced on long-axis view of the cine images when the atrium was at its minimum volume. In particular, the four-, three-, and two-chamber views were used to derive LA longitudinal strain. LA appendage and pulmonary veins were excluded from segmentation. After manual segmentation, the software automatically tracked the myocardial borders throughout the entire cardiac cycle. The quality of the tracking and contouring was visually validated and manually corrected by a radiologist with 4 years of experience in cardiac imaging. There are three peaks in the strain curve, including reservoir, conduit, and booster strain. Accordingly, their corresponding strain rate (SR) parameters were included.

### Machine learning

**Fig. 2:**
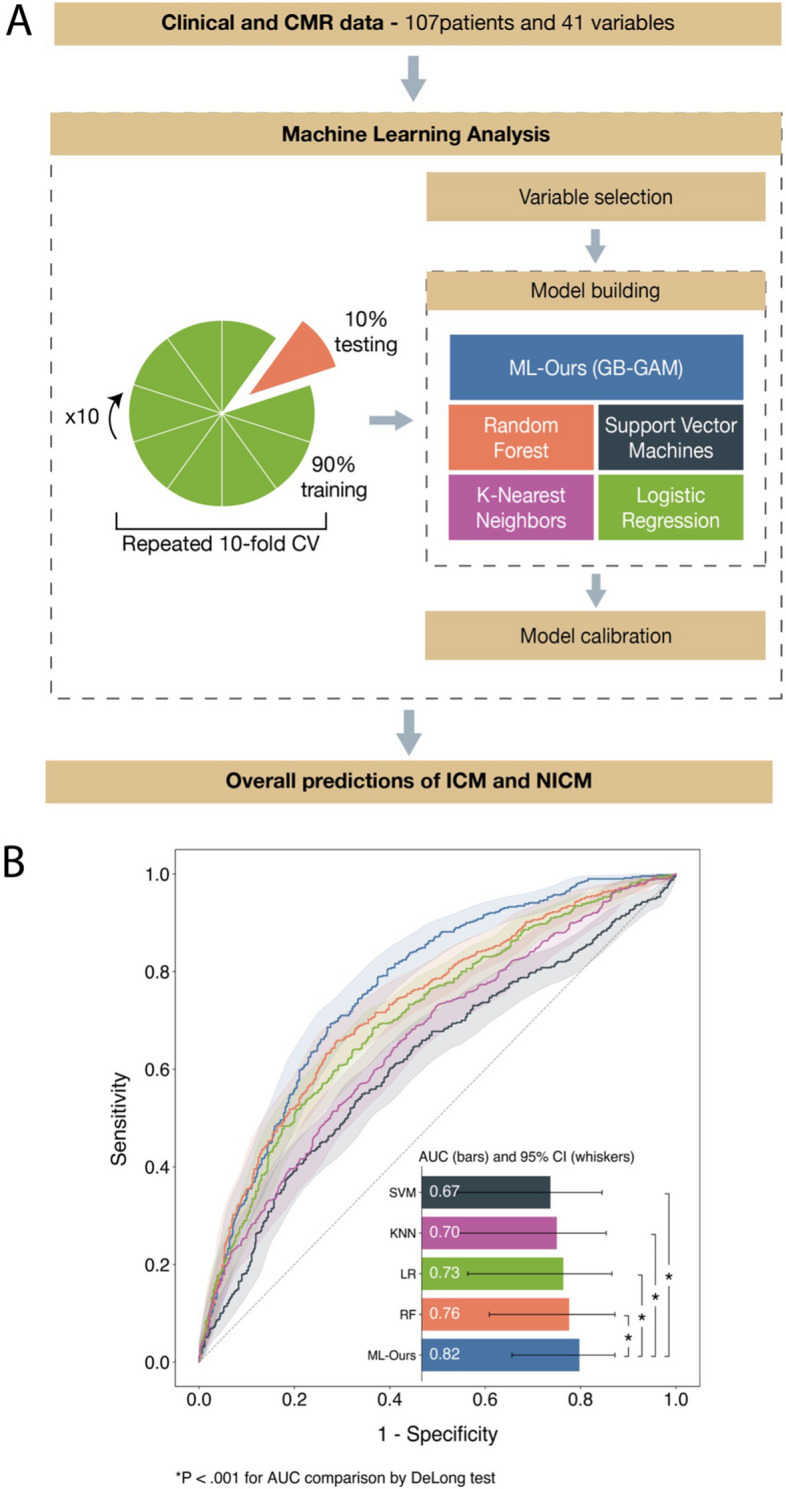
Overview of machine learning (ML) analysis and performance of ML models. **A** ML analysis involved automated variable selection by mean decrease in Gini impurity, derivation of our gradient boosting generalized additive model and traditional ML models, model calibration through isotonic regression, and 10 repetitions of the tenfold stratified cross-validation. **B** Receiver-operating characteristic curves reporting performance of ML models in identifying patients with ischemic cardiomyopathy when mixed with patients with non-ischemic cardiomyopathy. CV indicates cross-validation; GB-GAM, gradient boosting generalized additive model; SVM, support vector machine; KNN, k-nearest neighbors; LR, logistic regression and RF, random forest

Forty-one quantitative CMR-derived features of atrial strain (e.g., reservoir, conduit, booster), ventricular strain (e.g., longitudinal, radial, and circumferential strain of the ventricles), and ventricular function (e.g., ejection fraction, stroke volume) along with age, gender, and body surface area were available for feature selection and model building (see Supplemental Table 2 for all variables used in the analysis). Figure 2A shows an overview of the ML analysis. Briefly, it involved automated feature selection by Gini impurity reduction, model building using a gradient boosting generalized additive model (GB-GAM) and four traditional ML algorithms, probability calibration, and 10 repetitions of the tenfold stratified cross-validation (CV) for the entire process. No data were missing, and no pre-processing was applied to the data prior to model building.

### Variable selection

We used the mean decrease in Gini impurity method on all available features. We retained only features showing a *v* score at least 1.25 times greater than the average score (see Supplemental Methods for more details).

### Model definition

The GB-GAM algorithm was used to distinguish between ICM and NICM patients. This algorithm learns the relationships between each feature and the outcome separately using gradient boosting and combines them to produce a subject-level score that in this study is the log-odds of ICM. When predicting unseen patients, every feature value is used to index the corresponding learned feature functions to obtain a partial contribution. Then, these partial contributions are summed together to obtain the final predicted log-odds of ICM.

We compared its performance with four traditional ML algorithms, namely random forest (RF), support vector machines (SVM), k-nearest neighbors (KNN), and logistic regression (LR) (see Supplemental Methods for brief explanations). We refrained from tuning hyperparameters due to the small sample size and opted for settings derived from previous experiments (refer to Supplemental Methods for detailed hyperparameter values used for training).

### Probability calibration

Predicted probabilities were calibrated using an isotonic regression approach to obtain more reliable estimates of the true probabilities, thus improving the accuracy of downstream analysis [17] (see the Supplemental Methods for more details).

### Model training and testing

The entire ML process was executed in a tenfold stratified CV protocol with 10 repetitions, which allows for robust performance in small samples [18], and with a repeated leave-one-out procedure (see Supplemental Methods for detailed explanations).

### Variable importance and explanations of case examples

Variables were ranked by the average absolute impact on ML-predicted scores across all training subjects and the ten most impactful were further analyzed. Finally, we showed two examples of patient-level explanations of ML predictions with feature-specific values and contributions to the final prediction (more details in the Supplemental Methods).

### Diagnostic performance evaluation

We assessed the discrimination abilities of the ML models using both receiver operating characteristic (ROC) analysis and precision-recall curves, with area under the curve (AUC) and average precision as the respective metrics. Calibration was assessed both qualitatively through actual vs. predicted plots, and quantitatively with Brier scores. Additionally, sensitivity, specificity, F1 score, and positive and negative predictive value were calculated for the threshold that maximized the Youden’s J index (sensitivity + specificity − 1). Additional details on performance evaluation can be found in the Supplemental Methods section.

### Statistical analysis

For continuous variables, which were reported as the mean ± SD or median (inter-quartile range), we used the Shapiro–Wilk’s test to assess normality of residuals. Statistical comparisons between continuous variables for the ICM and NICM groups were performed using the *t*-test when normality of residuals was confirmed, while the Mann–Whitney *U* test was utilized in cases where normality was not established [18]. For categorical variables, which were reported as frequency (percent), we used the *χ*^2^ test. The DeLong test [19] was used to compare AUCs. Differences in sensitivities were tested with the McNemar test; the independent *t*-test was used to test for differences in average precisions and Brier scores. The outlined methodology was planned, and manuscript prepared according to the Checklist for Artificial Intelligence in Medical Imaging (CLAIM, see Supplemental Table 1) [20]. A *p* value < 0.05 was considered statistically significant (more details in Supplemental Methods).

## Results

### Study population

The baseline characteristics and CMR parameters of included patients are summarized in Tables 1 and 2. In summary, this retrospective study enrolled a total of 107 patients. Of those, 49 were ICM (34 males, mean age 60 ± 9 years) and 58 patients were NICM (38 males, mean age 56 ± 19 years). In patients with NICM, the etiologies included myocarditis (17, 29%), idiopathic dilated cardiomyopathy (20, 34%), Takotsubo cardiomyopathy (12, 20%), amyloidosis (5, %), and arrhythmogenic cardiomyopathy (4, 8%).

**Table 1:** Demographics characteristics, clinical risk factors, medications, and clinical presentation of the study population. Results are shown as mean ± SD, median (inter-quartile range), or *n* (%) as appropriate. *ICM* ischemic cardiomyopathy, *NICM* non-ischemic cardiomyopathy, *BSA* body surface area, *CAD* coronary artery disease, *ASA* acetylsalicylic acid, *ACE* angiotensin-converting enzyme inhibitors, *ARB* angiotensin receptor blocker, *PPI* proton pump inhibitors

	Total subjects	ICM	NICM	*p* value
Demographics				
Gender (male)	35 (33.0%)	15 (31.0%)	20 (34.0%)	.996
Age (years)	61.0 (50.5–69.5)	60.0 (54.0–68.0)	63.0 (46.0–73.0)	.873
BSA (m^2^)	1.8 ± 0.2	1.8 ± 0.2	1.8 ± 0.2	.289
Cardiovascular risk factors				
Hypertension	55 (51.0%)	28 (57.0%)	27 (47.0%)	.879
Dyslipidemia	29 (27.0%)	14 (29.0%)	15 (26.0%)	.999
Obesity	9 (8.0%)	6 (12.0%)	3 (5.0%)	.786
Smoke	33 (31.0%)	20 (41.0%)	13 (22.0%)	.377
Diabetes	16 (15.0%)	7 (14.0%)	9 (16.0%)	1.000
Familiarity for CAD	16 (15.0%)	8 (16.0%)	8 (14.0%)	.998
Medications				
ASA	39 (36.0%)	21 (43.0%)	18 (31.0%)	.808
Statins	29 (27.0%)	18 (37.0%)	11 (19.0%)	.374
Antiplatelet agent	23 (21.0%)	14 (29.0%)	9 (16.0%)	.612
Beta blocker	52 (49.0%)	22 (45.0%)	30 (52.0%)	.974
ACE-I/ARB	59 (55.0%)	27 (55.0%)	32 (55.0%)	1.000
Diuretics	16 (15.0%)	5 (10.0%)	11 (19.0%)	.808
Metformin	13 (12.0%)	5 (10.0%)	8 (14.0%)	.988
Insulin	2 (2.0%)	0 (0.0%)	2 (3.0%)	.787
PPI	16 (15.0%)	10 (20.0%)	6 (10.0%)	.715
Clinical presentation				
Chest pain	71 (66.0%)	40 (82.0%)	31 (53.0%)	.051
Heart failure	14 (13.0%)	2 (4.0%)	12 (21.0%)	.168
Arrhythmias	18 (17.0%)	7 (14.0%)	11 (19.0%)	.981

**Table 2:** CMR findings of the study population. Results are shown as mean ± SD, median (inter-quartile range) or *n* (%) as appropriate. *ICM* ischemic cardiomyopathy, *NICM* non-ischemic cardiomyopathy, *LV* left ventricle, *EDV* end-diastolic volume, *ESV* end-systolic volume, *SV* stroke volume, *BSA* body-surface area, *RV* right ventricle, *RS* radial strain, *CS* circumferential strain, *LS* longitudinal strain

	Total subjects	ICM	NICM	*p* value
CMR findings				
Reservoir (%)	20.0 (14.0 to 25.8)	19.8 (12.7 to 22.8)	21.4 (15.3 to 26.8)	.185
Reservoir rate (%)	0.9 (0.7 to 1.2)	0.8 (0.6 to 1.1)	0.9 (0.7 to 1.4)	.164
Conduit (%)	8.9 (4.8 to 12.6)	8.8 (4.4 to 11.8)	9.1 (5.7 to 14.8)	.289
Conduit rate (%)	− 0.9 (− 1.3 to − 0.6)	− 0.8 (− 1.1 to − 0.5)	− 0.9 (− 1.6 to − 0.7)	.085
Booster (%)	11.2 (7.8 to 14.0)	10.8 (7.8 to 12.7)	11.4 (7.6 to 14.1)	.411
Booster rate (%)	− 1.4 ± 0.6	− 1.4 ± 0.5	− 1.4 ± 0.6	.674
LVEF (%)	43.8 (31.6 to 48.3)	37.1 (26.4 to 49.3)	44.5 (38.2 to 47.7)	.091
Heart rate (BPM)	68.0 (63.0 to 77.0)	70.0 (63.0 to 77.0)	67.0 (61.2 to 76.8)	.485
LV mass (g)	116.6 (91.3 to 145.9)	127.5 (106.2 to 146.5)	107.0 (89.2 to 133.3)	.063
LVEDV / BSA (mL/m2)	95.5 (79.9 to 126.0)	107.0 (83.2 to 130.9)	90.2 (78.8 to 116.7)	.178
LVESV / BSA (mL/m2)	50.6 (39.1 to 82.6)	58.6 (45.0 to 94.6)	45.2 (35.3 to 66.5)	.013
LVSV / BSA (mL/m2)	39.6 (32.7 to 49.8)	37.8 (28.4 to 47.5)	43.7 (36.4 to 50.5)	.042
LV mass / BSA (g/m2)	64.0 (54.0 to 77.7)	67.8 (61.4 to 76.4)	60.8 (51.8 to 77.8)	.114
RVEF (%)	52.4 (45.6 to 58.9)	53.8 (48.3 to 60.0)	50.3 (44.0 to 57.8)	.168
RVEDV / BSA (mL/m2)	67.2 (54.3 to 86.3)	63.9 (51.7 to 78.7)	72.5 (61.0 to 89.3)	.018
RVESV / BSA (mL/m2)	32.2 (24.4 to 43.3)	30.3 (23.4 to 36.0)	35.2 (26.8 to 49.0)	.011
RVSV / BSA (mL/m2)	35.5 ± 12.9	33.7 ± 13.1	37.0 ± 12.7	.199
Basal LVRS (%)	21.9 (15.2 to 31.1)	17.9 (14.5 to 27.3)	24.1 (15.8 to 32.5)	.043
Basal LVCS (%)	− 14.4 ± 4.9	− 13.3 ± 4.8	− 15.2 ± 4.8	.044
Basal LVLS (%)	− 10.9 (− 14.0 to − 8.3)	− 10.6 (− 12.4 to − 7.8)	− 11.9 (− 15.0 to − 8.8)	.086
Mid LVRS (%)	18.2 (10.6 to 25.1)	15.6 (9.0 to 23.0)	21.4 (13.4 to 25.7)	.029
Mid LVCS (%)	− 12.8 (− 16.1 to − 8.4)	− 11.4 (− 14.5 to − 7.2)	− 14.3 (− 16.4 to − 10.2)	.018
Mid LVLS (%)	− 12.2 (− 15.7 to − 7.6)	− 9.9 (− 14.3 to − 6.2)	− 13.8 (− 16.4 to − 10.4)	.007
Apical LVRS (%)	22.3 (12.6 to 36.0)	14.9 (8.2 to 29.9)	25.9 (19.9 to 36.5)	.006
Apical LVCS (%)	− 14.8 (− 20.1 to − 9.6)	− 11.0 (− 17.6 to − 6.4)	− 16.2 (− 20.2 to − 13.7)	.004
Apical LVLS (%)	− 11.2 (− 15.6 to − 8.0)	− 9.6 (− 13.7 to − 6.8)	− 12.7 (− 15.7 to − 9.5)	.016
Global LVRS (%)	20.5 (12.6 to 28.6)	16.0 (10.4 to 26.9)	23.5 (15.6 to 29.0)	.024
Global LVCS (%)	− 13.4 ± 5.0	− 12.1 ± 5.2	− 14.4 ± 4.5	.015
Global LVLS (%)	− 11.4 (− 15.1 to − 7.8)	− 9.8 (− 13.4 to − 6.9)	− 12.9 (− 15.4 to − 8.9)	.014
Basal RVRS (%)	14.5 ± 6.7	14.0 ± 6.0	14.8 ± 7.2	.533
Global RVCS (%)	− 9.0 (− 11.2 to − 6.2)	− 9.2 (− 10.7 to − 5.8)	− 8.5 (− 11.9 to − 6.3)	.958
Mid RVRS (%)	19.2 (12.4 to 24.6)	20.0 (13.6 to 26.1)	19.0 (11.8 to 24.3)	.499
Mid RVCS (%)	− 11.8 ± 4.6	− 11.9 ± 5.1	− 11.7 ± 4.2	.813
Apical RVRS (%)	23.2 (15.8 to 35.0)	25.0 (16.0 to 37.0)	22.6 (15.3 to 33.4)	.548
Apical RVCS (%)	− 13.8 (− 18.7 to − 9.8)	− 15.1 (− 19.7 to − 9.6)	− 13.4 (− 18.1 to − 10.4)	.528
Global RVRS (%)	17.5 (12.2 to 22.2)	17.9 (12.6 to 21.5)	17.0 (11.1 to 23.1)	.738
Global RVCS (%)	− 10.7 ± 4.0	− 10.8 ± 4.0	− 10.6 ± 4.0	.810
Global RVLS (%)	− 16.4 (− 19.0 to − 12.4)	− 16.5 (− 20.0 to − 13.2)	− 16.1 (− 18.3 to − 11.1)	.224

### Performance of ML with repeated tenfold testing

**Fig. 3:**
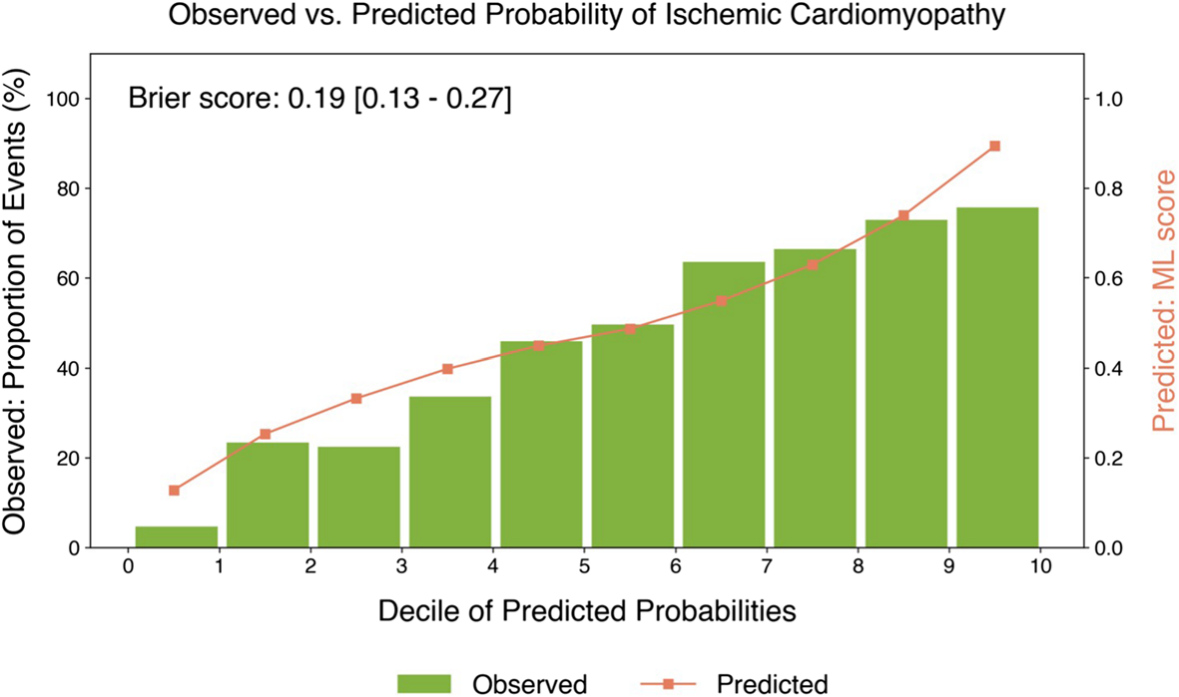
Calibration of the proposed ML model. Calibration plot after 10 repetitions of the tenfold cross-validation reporting a comparison between observed frequencies (green bars) and ML-predicted probabilities (orange curve) for ischemic cardiomyopathy, grouped by deciles of predicted probabilities

AUCs of ML models after 10 repetitions of the tenfold CV are shown in Fig. 2B. The proposed ML model (ML-Ours) exhibited a higher AUC compared with all other ML models in identifying ICM patients (ML-Ours 0.82 vs SVM 0.67, KNN 0.70, LR 0.73, RF 0.76, all *p* < 0.05). The average precision was also highest (ML-Ours 0.82 vs SVM 0.72, KNN 0.67, LR 0.78, RF 0.80, *p* < 0.05 vs SVM and KNN, *p* = 0.10 vs LR, *p* = 0.47 vs RF) (Supplemental Fig. 1). Using the threshold that maximizes the Youden’s J index as the optimal cutoff to classify patients as ICM, the proposed ML model showed the highest sensitivity (72%, 95% CI [68–76], *p* < 0.05 vs all) and a specificity of 68% (95% CI [64–71]). At 90% sensitivity, the specificity was 43% (95% CI [39–47]). Furthermore, ML-Ours exhibited the highest F1 score (0.69 [0.65–0.72]) and area under the precision-recall curve (0.82 [0.50–1.00]). For a comprehensive overview of performance scores from repeated tenfold testing, please see Supplemental Table 3. The current approach also showed good agreement between ML-predicted and observed probabilities of ICM, as confirmed by the calibration plot (Fig. 3) and lowest Brier score (0.19, 95% CI [0.13–0.27], *p* < 0.05 vs SVM, KNN, and LR, *p* = 0.06 vs RF).

### Performance of ML with repeated leave-one-out testing

After conducting 10 repetitions of the leave-one-out testing, the proposed ML-Ours model demonstrated a consistently higher AUC compared to all other traditional ML models (ML-Ours 0.73 vs. SVM 0.59, KNN 0.62, and RF 0.69, *p* < 0.05; LR 0.68, *p* = 0.07) (Supplemental Fig. 2). Although the improvements in AUC were not always statistically significant, the relative performance of the model remained consistent with the results obtained through repeated tenfold CV. Furthermore, the proposed approach exhibited the highest sensitivity (0.66 [0.61–0.70]) and F1 score (0.64 [0.61–0.67]). For a comprehensive overview of performance scores from repeated leave-one-out testing, please see Supplemental Table 4.

### Computational complexity

The experimental outcomes are achieved for the proposed structure using Python version 3.9.6 on an Apple M1 Max with 10 cores, 64 GB RAM, 48 MB Cache, and integrated GPU under a 64-bit operating system. In the repeated tenfold CV setting, the outer CV scheme partitions the available 107 subjects in train and testing folds of approximately 96 and 11 subjects, respectively. Of these 96, 15% is reserved for calibration purposes, leading to approximately 82 subjects for training and 15 for calibration. On average, feature selection over fivefold CV required 0.312 ± 0.02 s; training of the proposed approach required 22.7 ± 11.9 s; model calibration required 0.0025 ± 0.0002 s; and prediction of 11 test subjects required 0.0013 ± 0.0002 s (Supplemental Fig. 3A).

In the leave-one-out testing scheme, 106 of 107 are used for training and 1 is held out as test. Of these 106, 15% (approximately 16 subjects) is held out for model calibration, leading to 90 subjects for training. On average, feature selection over fivefold CV required 0.31 ± 0.02 s; training of the proposed approach required 14.3 ± 11.8 s; model calibration required 0.0025 ± 0.0003 s; and prediction of 1 test subject required 0.0013 ± 0.0003 s (Supplemental Fig. 3B).

### Important features for identifying ICM

**Fig. 4:**
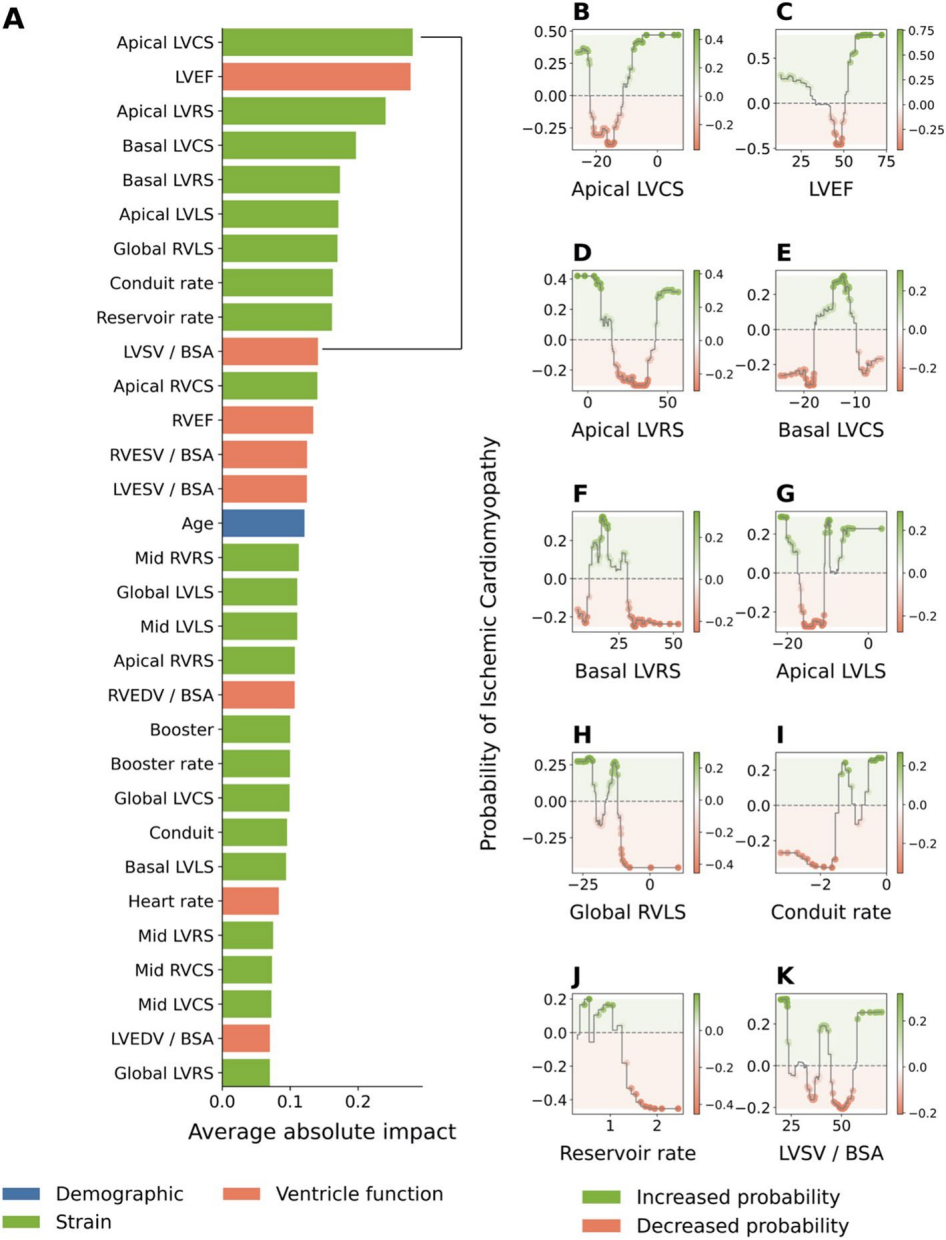
Impact-ranking of variables in identifying patients with ischemic cardiomyopathy. **A** Variables were ranked by their average absolute impact on machine learning (ML) predictions of ischemic cardiomyopathy (ICM). This figure shows the average absolute contributions to ML-predicted probability of ICM of selected features from one representative fold of the cross-validation procedure (full details on the variables used in the Supplemental Appendix). **B**–**K** Detailed relationship between the top ten most impactful features and the predicted probability of ICM. Different values within the range of possible values that each feature can take on have a different impact on the predictions: values in green regions have a positive impact on predictions (these values increase the probability of ICM) whereas values in orange regions have a negative contribution (they decrease the probability of ICM). LV indicates left ventricular; RV, right ventricular; LVCS, LV circumferential strain; LVEF, LV ejection fraction; LVRS, LV radial strain; LVLS, LV longitudinal strain; LVSV, LV stroke volume; BSA, indexing by body surface area; RVCS, RV circumferential strain; RVEF, RV ejection fraction; RVESV, RV end-systolic volume; LVESV, LV end-systolic volume; RVEDV, RV end-dyastolic volume; LVEDV, LV end-dyastolic volume; RVRS and RV radial strain

Both atrial and ventricular strain CMR parameters were important for the identification of ICM patients (Fig. 4A). Overall, left ventricle RS and CS, left ventricle ejection fraction (LVEF), right ventricle LS, conduit rate, and reservoir rate had the most impact on identifying ICM patients. Detailed contribution to the prediction of ICM by the top ten important variables is shown in Fig. 4B to K.

Greater basal and apical CS, right ventricle LS, and conduit rate values had more significant impact in discriminating ICM patients. Conversely, lower basal and apical RS, LVEF, and reservoir rate parameters had more impact on identifying ICM patients.

### Individualized explanations of ML predictions

**Fig. 5:**
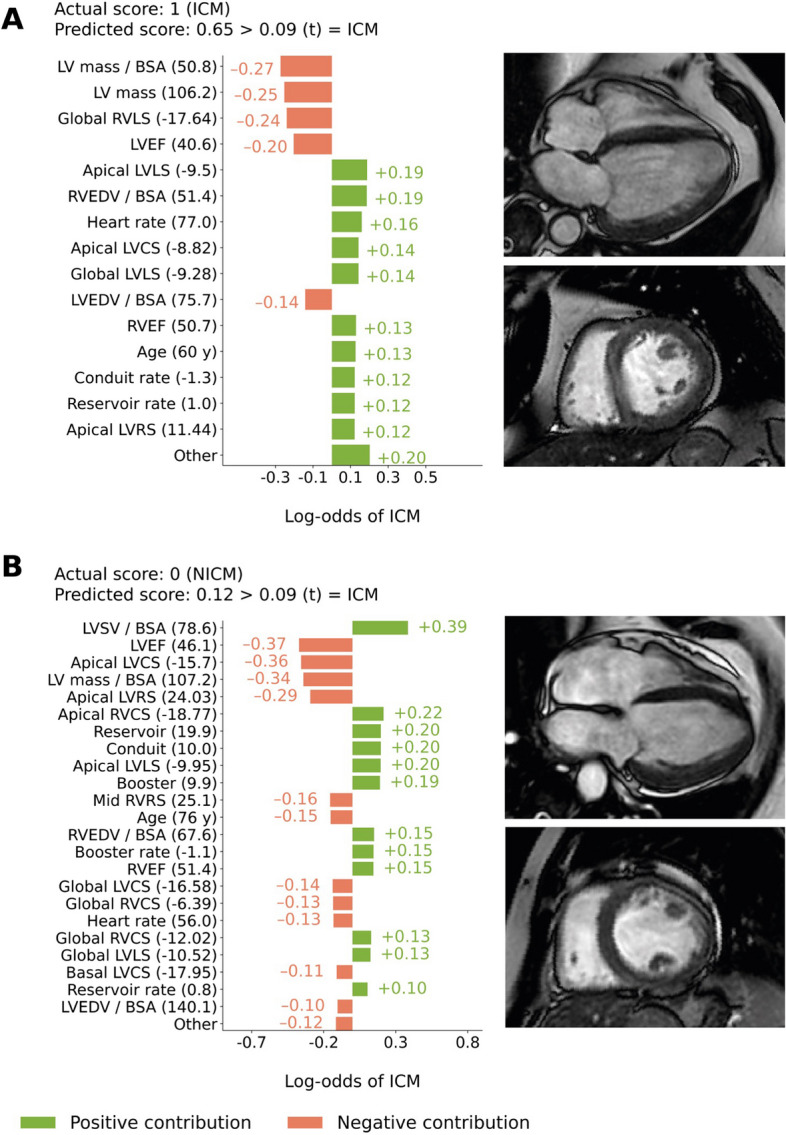
Explanations of machine learning (ML) predictions with subject-specific feature contributions. This figure shows two case examples from a representative fold of the cross-validation procedure.** A** A 60-year-old male with ischemic cardiomyopathy (ICM) correctly identified as having ICM by the proposed ML model. **B** A 76-year-old female with non-ischemic cardiomyopathy wrongly classified by the proposed model as ICM (false positive). On the left, individual contributions of each variable to the predicted log-odds of ICM for each patient (*x*-axis) are shown, along with the value of each variable. Green and orange bars indicate positive and negative contributions towards a prediction of ICM, respectively. For each case, the predicted score (i.e., the probability obtained after transforming the log-odds) and the threshold used for classification are reported for reference. On the right, cine CMR images in the long axis and the short axis are reported. Abbreviations as in Fig. 4

To understand better the decisional process of the proposed ML model when classifying unseen patients, Fig. 5 shows two case examples of patient-level explanations and feature-specific contributions to the predicted ML score. The first case (Fig. 5A) is a 60-year-old female with ICM correctly classified as ICM by the model. The second case is a 76-year-old female with NICM which was wrongly classified as ICM by the model (i.e., false positive). The *x*-axis reports the predicted probability of the patient having ICM; the *y*-axis reports the specific values of each covariate for that patient. The horizontal bars represent the influence of each feature on the overall prediction (log-odds of ICM), along with the specific increase (green bars) or decrease (orange bars) in the predicted log-odds.

## Discussion

The present study demonstrated that a ML algorithm trained with CMR left ventricular, left atrial, and right ventricular quantified strain and general cardiac functions could distinguish patients with ICM accurately from those with patients with NICM.

The major advantage of this novel ML approach is obviating the need for contrast media administration thus enhancing patient tolerability and shortening scan time at a lower cost.

In clinical practice, the exponential increase in CMR examinations may necessitate the optimization of clinical workflows with faster and more cost-effective CMR protocols. Furthermore, individuals who are ineligible for contrast media administration and have a limited tolerance for CMR examinations may find shorter, non-contrast CMR examinations advantageous. Non-contrast CMR imaging improves the applicability of CMR examinations and offers greater patient comfort and can lower costs [12].

Several authors have explored the diagnostic capability of non-contrast cine-CMR images using ML-based models as an alternative to LGE images [11, 13, 21–23]. Avard et al explored a ML algorithm and radiomics features to differentiate myocardial infarction and normal cases on non-contrast cine-CMR images showing that the AI-based model yielded optimal results with an AUC of 0.93 [10]. Similarly, Larroza et al utilized a support vector machine classifier to explore the potential of texture analysis with cine-CMR images in distinguishing between infarcted nonviable, viable, and remote segments. The results showed that texture analysis could effectively detect non-viable segments on non-contrast cine-CMR images, achieving an impressive AUC of 0.849 and a sensitivity of 92% [11]. Conversely, Zhang et al evaluated virtual native enhancement combining cine-CMR images and native T1 mapping to produce LGE-like images. This approach used a deep learning model achieving a strong correlation with LGE in quantifying scar size and transmurality with an accuracy, specificity, and sensitivity of 84%, 100%, and 77%, respectively [12].

Previous ML-based cine-CMR research focused on the application of radiomics analysis, which requires time-consuming quantitative post-processing image analysis or the acquisition of additional CMR sequences, leading to longer CMR examinations.

To the best of our knowledge, this is the first work focused on a ML-based model that includes cine-CMR parameters to discriminate between ICM and NICM. Previous work investigating myocardial strain-derived parameters to determine the etiology of heart failure have focused on the analysis of a single cardiac chamber or on radiomics analysis [10, 13, 23–25]. The present study has a retrospective study design and a single-center data collection approach. The retrospective analysis enables to harness a substantial dataset of CMR cases, encompassing a significant timeframe, which is essential for training and validating a robust ML model. Additionally, the decision to focus on a single center offered a dataset characterized by consistency and homogeneity in relation to imaging protocols and CMR scanner which contributes to reducing potential variability and confounding factors.

The current study simultaneously analyzed multiple cardiac chambers using an ML algorithm. The physiological “communication” between cardiac chambers may be too complex to be captured using the traditional regression models. A ML-based model for assessing the relationship between cardiac chambers interaction can overcome some limitations of common regression techniques.

In the features importance analysis, ventricular and left atrial strain parameters presented the most discriminative value in discriminating between ICM and NICM.

In fact, NICM tends to show global myocardial fiber dysfunction involving both ventricles in comparison with ICM that shows a more regional dysfunction [26]. The contribution of circumferential and radial strain parameters in discriminating ICM from NICM seen in the current study is explained by the different effects on subepicardial and transmural fiber by myocyte injuries.

Indeed, the myocardium is composed of three layers of fibers, namely (1) subendocardial fibers acting on longitudinal shortening, (2) subepicardial fibers acting on circumferential shortening, and (3) transmural fibers acting on radial shortening [27, 28].

The pathophysiology of myocardial infarction is characterized by a waveform appearance from the subendocardium to the epicardial layer. Therefore, the subendocardial fibers are the earliest myocardium layers involved in ischemia [28, 29]. In addition, it has been suggested that impairment in the contraction of longitudinal fiber in ICM is compensated by the augmentation of the other layers [29, 30]. Similarly, the RV strain parameters’ impact in discriminating between ICM and NICM demonstrated in the present study may relate to the ventricular interaction through the interventricular septum [31]. Indeed, the RV shares oblique fibers with the LV in the interventricular septum. In the RV contraction, the oblique septal fibers are more efficient than the free wall transverse fibers and consequently, LV remodeling leads to septal fibers dysfunction impairing RV contraction [32].

Another hypothesis suggested is an intrinsic injury of the RV myocardium in NICM patient [33]. Due to the anatomical connection of the ventricle and atrium, their contribution to differentiate ICM and NICM is expected in this model. The LA strain parameters more relevant in the proposed model are reservoir and conduit strain rate parameters reflecting LA expansibility and stiffness.

In patients with LV dysfunction, LA contraction rises to maintain optimal LV filling. Consequently, early in ventricular dysfunction LA pump function is increased but LA stiffness augmented, and work mismatch occurs. Subsequently, LA pump function decreases as a result of the progression of LV dysfunction due to the increased afterload imposed on the LA [7, 34]. In addition, some authors suggested a direct myopathic involvement of the LA myocardium in various NICM [22, 35, 36].

### Practical advantages

The aim of the current study was to investigate the capability of an ML algorithm using cardiac function, volumes, and atrial and ventricular strain features from cine-CMR in discriminating ischemic from non-ischemic etiologies. Using cine-CMR features could potentially avoid the contrast media administration and increase the clinical availability of CMR examinations allowing increased accessibility to CMR examinations achieved through reduced costs and faster imaging acquisition.

### Limitations

The following study limitations should be acknowledged and addressed in future research before the presented method can be employed in clinical practice. First, the relatively small sample size was evaluated with a single CMR scanner and the retrospective single-center observational design of the study with no external validation dataset. Although we have taken precautions to mitigate the challenges posed by the limited dataset, the sample size and single-center study design could potentially impact the generalizability to more diverse populations, encompassing variations in race, medications, and other factors. To confirm the robustness of the present findings, future multi-center studies are prompted to facilitate the incorporation of larger, more varied datasets, bolstering the reliability and broader applicability of the proposed approach. Even though the purpose of the study was to evaluate an AI model to discriminate between ICM and NICM, and thus required a heterogenous NICM cohort, the composition of the evaluated population might be influenced by statistical variations as may the subsequent results.

Second, although we have taken precautions when training models and estimating generalization performance, this model may still suffer from overfitting. To guard against overfitting, we employed cross-validation to select features with diagnostic value, train algorithms, calibrate predicted probabilities, and evaluate performance on the same cohort, by considering non-overlapping subsets of the data and thus reducing the bias in performance estimation.

Finally, in the current study, the predictive value of strain and parametric mapping parameters for adverse cardiovascular events was not assessed at follow-up. The promising results could prompt further prospective trials including a larger number of patients to confirm the present findings. However, implementing non-contrast AI models into real-world clinical practices poses considerable challenges, including the lack of transparency and interpretability of AI models, the needed of a large amount of annotated data from different centers, as well as ethical and legal issue. The derivation of an effective non-contrast ML model that can be applied to the real-world clinical practice would require a considerably larger training group across different centers and patient groups and its validation would require an independent validation cohort. Furthermore, the development of a privacy protection algorithm is essential, which should integrate encryption and AI techniques to achieve secure and generalizable non-contrast AI models.

## Conclusion

The proposed GB-GAM model integrating multi-chamber myocardial strain, function, and volumes on non-contrast CMR scan achieved a competitive diagnostic accuracy (AUC = 0.82) with a sensitivity of 0.72 and a specificity of 0.68 in discriminating between ICM and NICM. Non-contrast AI models may offer clinical benefits to CMR examinations by reducing costs and scan times, enhancing availability and broadening clinical applicability.

### Supplementary Information

Below is the link to the electronic supplementary material.

Supplementary file1 (PDF 1117 KB)

## Data Availability

All code used for running experiments, deriving models, and producing the figures is available on a GitHub repository at https://github.com/francescopisu/ICM_NICM_ML. We have also used Zenodo to assign a DOI to the repository: 10.5281/zenodo.8279355. All future updates will be made available both to the GitHub repository and to the Zenodo.

## Abbreviations

CMR: Cardiovascular magnetic resonance
CV: Cross-validation
CVDs: Cardiovascular diseases
GB-GAM: Gradient boosting generalized additive model
ICM: Ischemic cardiomyopathy
LA: Left atrium
LGE: Late-gadolinium enhancement
LV: Left ventricle
LVEF: Left ventricle ejection fraction.
ML: Machine learning
NICM: Non-ischemic cardiomyopathy
RV: Right ventricle

## Declarations

### Guarantor

The scientific guarantor of this publication is the corresponding author.

### Statistics and biometry

One of the authors has significant statistical expertise.

### Study subjects or cohorts overlap

None of the article contents have been published previously.

### Methodology

• retrospective

• diagnostic or prognostic study

• performed at one institution
