# Supplementary material for: Cine-cardiac magnetic resonance to distinguish between ischemic and non-ischemic cardiomyopathies: a machine learning approach
Source: Eur Radiol. 2024 Mar 7;34(9):5691–704. doi: 10.1007/s00330-024-10640-8 (PMC11364683; doi:10.1007/s00330-024-10640-8)
Supplement: Supplementary file 1 — Supplementary file1 (PDF 1117 KB) [file 330_2024_10640_MOESM1_ESM.pdf]

Cine-Cardiac Magnetic Resonance to distinguish between ischemic and non-ischemic cardiomyopathies: a machine learning approach.

Content	Page number
Supplemental Methods	2
Supplemental Figure Legends	5
Supplemental Table 1. CLAIM checklist for Artificial Intelligence in Medical Imaging.	6
Supplemental Table 2. Description of variables used for machine learning.	8
Supplemental Table 3. Diagnostic performance of the proposed approach and traditional ML models.	10
Supplemental Table 4. Diagnostic performance of our approach and traditional ML models from repeated leave-one-out testing.	11
Supplemental Figure 1. Precision-recall curves of the proposed approach and traditional ML models.	12
Supplemental Figure 2. Receiver-operating characteristic curves of the proposed approach and traditional ML models from repeated leave-one-out testing.	13
Supplemental Figure 3. Time required for the entire model building procedure by the proposed approach and traditional ML models.	14
References	15

Eur Radiol (2024) Cau R, Pisu F, Pintus A et al

Supplemental Methods

Variable Selection

We used a random forest algorithm to rank the variables, based on their mean Gini impurity decrease within each tree of the forest. We first fitted a random forest using all available variables in each fold of the 5-fold cross-validation (CV), and then selected only those attributes with an importance score at least 1.25 times greater than the average importance score. We then used the union of the five resulting feature sets for our ML analysis. Gini impurity index ranges from 0 to 0.5, where a lower value indicates better performance. This index reflects the probability of misclassification if a randomly selected element from a set were labeled according to the target distribution of that set's elements.

The choice of using the Gini impurity for variable selection is well-justified for several reasons. Firstly, in our dataset containing approximately half the number of features as compared to the subjects, multicollinearity is a potential concern. By leveraging Gini impurity, the features with most impact on detecting ICM can be identified and prioritized, thereby mitigating the impact of multicollinearity, and promoting feature diversity. Secondly, Gini impurity facilitates the identification of non-linear relationships between the variables and the target class. Lastly, in limited sample size scenarios, outliers may significantly influence the analysis. The use of Gini impurity provides greater robustness to outliers compared to other traditional metrics, ensuring more reliable and stable variable selection outcomes.

Model Definition

We employed the tree-based gradient-boosting generalized additive model (GB-GAM) to build a model for distinguishing between ICM and NICM. In gradient boosting, a type of ensemble learning, multiple estimators – typically decision trees – are trained sequentially, by adjusting weights of data points to penalize misclassifications at each iteration, to produce an estimator with greater generalization performance[1].

The GB-GAM model is explainable-by-design as it learns a relationship between each feature and the log-odds of the outcome separately. When testing on unseen data, the model considers every patient-specific feature value and indexes the learned feature functions to obtain the relative contribution of that feature to the predicted log-odds. Then, it sums all these contributions to obtain the final predicted log-odds of ICM, which is then transformed to a probability score between 0 and 1. GB-GAM is also capable of discovering relevant pairwise feature interactions and integrating it into the final model with complete transparency and interpretability.

In greater detail, the GB-GAM algorithm starts by discretizing all continuous variables into a specific number of bins. The discretization simplifies the model and enhances its interpretability. Then, a standard additive model or initial model is fitted to model the non-linear relationships between each individual binned feature and the response variable. In the GB-GAM algorithm, shallow regression trees are used to represent these smooth functions. The initial model, which is a linear combination of smooth functions of the individual features or the learned separate models, is used as a starting point for the gradient boosting process, which adds shallower tree-like models to capture the pairwise interactions between features. After fitting the initial model, the algorithm focuses on pairwise interactions. For this matter, the residuals of each individual feature are calculated by subtracting the predicted values for that feature of the initial model from the actual binned values; these residuals represent the part of the response variable that is not explained by the initial model. Then, for each pair of binned features, a shallow tree-like model is fitted to the residual of one feature with respect to the other, and viceversa; in other words, it uses the residuals of the former to predict the residuals of the latter. This effectively captures the pairwise interactions

between the two features in both directions. Finally, the predicted values of the two separate models are multiplied to obtain a single value representing the pairwise interaction.
These residuals will be then used to fit the next set of shallow models in the gradient boosting process, which will attempt to capture the remaining information in the data that was not captures by the previous models. The predictions of these new models are added to the predictions of the previous models to obtain updated predictions for the response variable. The process will continue until a stopping criterion is met. The final model is a sum of the initial models and the tree-like models that were added during the gradient boosting process, and it captures both the main effects of each individual feature and the pairwise interactions between features.[2]
We compared the performance of the GB-GAM model with four traditional machine learning (ML) algorithms, for which a brief description will follow.
Random forests is a supervised, tree-based ensemble that uses bootstrap aggregating (also known as bagging) to train multiple decision trees and combine their predictions to produce a stronger classification[3]. Support vector machines are linear classifiers based on the maximum margin concept; in binary classification settings, it searches the hyperplane that maximally separates data points of the two classes[4]. K-nearest neighbors is an algorithm that does not require explicit training, and which makes predictions on-the-fly based on a similarity measure calculated considering the k-nearest neighbors of each new data point to be classified[5]. Finally, logistic regression is a statistical method that models the expected value of a target binary variable conditional on the value of one or more features[5].

The following hyperparameters were used when training the models:

- **Explainable Boosting Classifier:**
  - Interactions: 15
  - learning_rate: 0.02
  - min_samples_leaf: 5,
  - outer_bags: 35,
  - inner_bags: 35,
  - max_bins: 128,
  - max_leaves: 3
- **Logistic Regression:**
  - penalty: elasticnet,
  - solver: saga,
  - l1_ratio: 0.3,
  - max_iter: 10000
- **Support Vector Machines:**
  - C: 1.0,
  - kernel: rbf,
  - gamma: scale,
  - probability: True
  - class_weight: balanced
- **K-nearest Neighbors:**
  - n_neighbors: 5,
  - weights: uniform,
  - algorithm: auto,
  - leaf_size: 30,
  - p: 2,
  - metric: minkowski

Probability Calibration

Briefly, a non-decreasing function is fitted to predicted probabilities so that each new prediction for the training data is closest to the targets in terms of mean squared error.

Model Training and Testing

Tenfold CV has several advantages compared to the classic split-sample approach: 1) data for training and testing is maximized, whilst avoiding overlap between data used for training models and evaluating performance and reducing risk of overfitting; 2) it reduces the bias when estimating predictive ability of models and 3) reduces the variance when estimating the generalization error[6]. Briefly, the available data is divided in ten equally sized partitions, each containing similar proportions of ICM and NICM. Then, 9/10th of the data was used for feature selection and model training, and the remaining 1/10th was used for testing. This protocol ensures each data point is used for evaluation once and only once, and that the model is always evaluated on previously unseen patients.
For the leave-one-out testing, N-1 subjects were utilized for feature selection, calibration, and model building, while the remaining subject served as the test case. This procedure was then iterated for every subject in the cohort, ensuring each was independently evaluated as the test case. To increase robustness, we repeated the entire procedure 10 times, employing dataset shuffling in each iteration to create different partitions for feature selection and calibration.
The proposed model produced a continuous score from 0 to 1 representing the likelihood of ICM for all subjects in each testing fold. These scores were then concatenated and used to estimate average performance in unseen data.

Variable Importance and Explanations of Case Examples

We performed the ranking based on average absolute impact on ML predictions separately for each cross-validation testing subset. The ten most impactful variables were further analyzed by relating feature values with the ML-predicted log-odds of ICM.

Diagnostic Performance Evaluation

For leave-one-out (LOO) testing, out-of-fold predictions are obtained pooling together predictions of the independent test case kept aside for LOO cross-validation (CV) fold. After one iteration of LOOCV, 119 predictions are obtained and used to measure diagnostic performance of models. The entire procedure is repeated 10 times, each time pooling out-of-fold predictions. After 10 repetitions, the sets of 10 performance measures are used to compute median and 95% confidence intervals using the percentile method for probability-based measures; for metrics computed using binary labels after Youden’s J index-based dichotomization, the bootstrap method with 5000 iterations was used on accumulated ground-truths and predicted probabilities over 119 predictions and 10 repetitions, for a total of 1190 predictions.
Confidence bands around curves were computed using the bootstrap method with 5000 iterations. Confidence intervals (CI) at 95% confidence level for probability-based measures were calculated using the percentile method. These CI were computed based on the scores obtained from ground-truths and predictions across all 10-fold CV repetitions. CI for sensitivity, specificity, F1 score, positive predictive value and negative predictive value were calculated with the bootstrap method on ground-truths and predicted probabilities accumulated across all CV folds.

Statistical Analysis

We used the Python implementation of the GB-GAM algorithm provided by the *interpret* framework. All statistical analyses were performed with the R software (R Foundation for Statistical Computing, Vienna, Austria, version 4.1.0) and the Python language (Python Software Foundation. Python Language Reference, version 3.9).

Supplemental Figure Legends

Supplementary Figure 1. **Precision-recall curves of the proposed approach and traditional ML models**. For each model is reported the average precision after 10 repetitions of 10-fold cross-validation. Median value is annotated on the left side of each horizontal bar. Horizontal whiskers indicate 95% confidence intervals. Vertical whiskers indicate comparisons between models and are annotated with P-values by the independent t-test.

Supplemental Figure 2. **Receiver-operating characteristic curves of the proposed approach and traditional ML models from repeated leave-one-out testing**. For each model is reported the area under the receiver-operating characteristic after 10 repetitions of the leave-one-out testing. Median value is annotated on the left side of each horizontal bar. Horizontal whiskers indicate 95% confidence intervals. Vertical whiskers indicate comparisons between models and are annotated with P-values by the DeLong test.

Supplemental Figure 3. Time required for the entire model building procedure by the proposed approach and traditional ML models. A) Average time from repeated leave-10-out testing and B) from repeated leave-one-out testing represented by solid bars, with whiskers representing standard deviation. The time needed depends on the feature selection, training and calibration and prediction (stacked bars).

Supplemental Tables

Supplemental Table 1. CLAIM checklist for Artificial Intelligence in Medical Imaging

Section / Topic	No.	Item	
TITLE / ABSTRACT			
	**1**	Identification as a study of AI methodology, specifying the category of technology used (e.g., deep learning)	✓
	**2**	Structured summary of study design, methods, results, and conclusions	✓
INTRODUCTION			
	**3**	Scientific and clinical background, including the intended use and clinical role of the AI approach	✓
	**4**	Study objectives and hypotheses	✓
METHODS			
Study Design	**5**	Prospective or retrospective study	✓
	**6**	Study goal, such as model creation, exploratory study, feasibility study, non-inferiority trial	√
Data	**7**	Data sources	✓
	**8**	Eligibility criteria: how, where, and when potentially eligible participants or studies were identified (e.g., symptoms, results from previous tests, inclusion in registry, patient-care setting, location, dates)	✓
	**9**	Data pre-processing steps	✓
	**10**	Selection of data subsets, if applicable	**N/A**
	**11**	Definitions of data elements, with references to Common Data Elements	**N/A**
	**12**	De-identification methods	**N/A**
	**13**	How missing data were handled	**N/A**
Ground Truth	**14**	Definition of ground truth reference standard, in sufficient detail to allow replication	✓
	**15**	Rationale for choosing the reference standard (if alternatives exist)	✓
	**16**	Source of ground-truth annotations; qualifications and preparation of annotators	**N/A**
	**17**	Annotation tools	**N/A**
	**18**	Measurement of inter- and intrarater variability; methods to mitigate variability and/or resolve discrepancies	**N/A**
Data Partitions	**19**	Intended sample size and how it was determined	**N/A**
	**20**	How data were assigned to partitions; specify proportions	✓
	**21**	Level at which partitions are disjoint (e.g., image, study, patient, institution)	✓
Model	**22**	Detailed description of model, including inputs, outputs, all intermediate layers and connections	✓
	**23**	Software libraries, frameworks, and packages	✓

	**24**	Initialization of model parameters (e.g., randomization, transfer learning)	**N/A**
Training	**25**	Details of training approach, including data augmentation, hyperparameters, number of models trained	✓
	**26**	Method of selecting the final model	✓
	**27**	Ensembling techniques, if applicable	**N/A**
Evaluation	**28**	Metrics of model performance	✓
	**29**	Statistical measures of significance and uncertainty (e.g., confidence intervals)	✓
	**30**	Robustness or sensitivity analysis	✓
	**31**	Methods for explainability or interpretability (e.g., saliency maps), and how they were validated	✓
	**32**	Validation or testing on external data	**N/A**
RESULTS			
Data	**33**	Flow of participants or cases, using a diagram to indicate inclusion and exclusion	✓
	**34**	Demographic and clinical characteristics of cases in each partition	✓
Model performance	**35**	Performance metrics for optimal model(s) on all data partitions	✓
	**36**	Estimates of diagnostic accuracy and their precision (such as 95% confidence intervals)	✓
	**37**	Failure analysis of incorrectly classified cases	✓
DISCUSSION			
	**38**	Study limitations, including potential bias, statistical uncertainty, and generalizability	✓
	**39**	Implications for practice, including the intended use and/or clinical role	✓
OTHER INFORMATION			
	**40**	Registration number and name of registry	**N/A**
	**41**	Where the full study protocol can be accessed	**N/A**
	**42**	Sources of funding and other support; role of funders	✓

Mongan J, Moy L, Kahn CE Jr. Checklist for Artificial Intelligence in Medical Imaging (CLAIM): a guide for authors and reviewers. Radiol Artif Intell 2020; 2(2):e200029. https://doi.org/10.1148/ryai.2020200029

Supplemental Table 2. Description of variables used in machine learning. CAD indicates coronary heart disease; IPH, intraplaque hemorrhage.

**Name**	**Values**	**Description**
Age	continuous; years	The age of the patient
Gender	binary; 0/1	Gender of the patient; 0 = male, 1 = female
BSA	continuous; m^2	Body surface area
HR	continuous; BPM	Heart rate
LVEF	continuous; %	Left ventricular ejection fraction
LV MASS	continuous; g	Left ventricular mass index
LV MASS / BSA	continuous; g/m^2	Left ventricular mass indexed by BSA
LVEDV / BSA	continuous; ml/m^2	End-diastolic volume of left ventricle index by BSA
LVESV / BSA	continuous; ml/m^2	End-systolic volume of left ventricle index by BSA
LVSV / BSA	continuous; ml/m^2	Stroke volume of left ventricle index by BSA
RVEF	continuous; %	Right ventricular ejection fraction
RVEDV / BSA	continuous; ml/m^2	End-diastolic volume of right ventricle index by BSA
RVESV / BSA	continuous; ml/m^2	End-systolic volume of right ventricle index by BSA
RVSV / BSA	continuous; ml/m^2	Stroke volume of right ventricle index by BSA
Reservoir	continuous; %	Left atrium reservoir
Reservoir rate	continuous; %	Left atrium reservoir rate
Conduit	continuous; %	Left atrium conduit
Conduit rate	continuous; %	Left atrium conduit rate
Booster	continuous; %	Left atrium booster
Booster rate	continuous; %	Left atrium booster rate
Basal RVRS	continuous; %	Basal radial strain of right ventricle
Mid RVRS	continuous; %	Mid radial strain of right ventricle
Apical RVRS	continuous; %	Apical radial strain of right ventricle
Global RVRS	continuous; %	Global radial strain of right ventricle
Basal RVCS	continuous; %	Basal circumferential strain of right ventricle
Mid RVCS	continuous; %	Mid circumferential strain of right ventricle
Apical RVCS	continuous; %	Apical circumferential strain of right ventricle

Global RVCS	continuous; %	Global circumferential strain of right ventricle
Global RVLS	continuous; %	Global longitudinal strain of right ventricle
Basal LVRS	continuous; %	Basal radial strain of left ventricle
Mid LVRS	continuous; %	Mid radial strain of left ventricle
Apical LVRS	continuous; %	Apical radial strain of left ventricle
Global LVRS	continuous; %	Global radial strain of left ventricle
Basal LVCS	continuous; %	Basal circumferential strain of left ventricle
Mid LVCS	continuous; %	Mid circumferential strain of left ventricle
Apical LVCS	continuous; %	Apical circumferential strain of left ventricle
Global LVCS	continuous; %	Global circumferential strain of left ventricle
Basal LVLS	continuous; %	Basal longitudinal strain of left ventricle
Mid LVLS	continuous; %	Mid longitudinal strain of left ventricle
Apical LVLS	continuous; %	Apical longitudinal strain of left ventricle
Global LVLS	continuous; %	Global longitudinal strain of left ventricle

Supplemental Table 3. Diagnostic performance of the proposed approach and traditional ML models. Results are shown as median (95% confidence intervals [CI]). CIs for probability-based metrics (ROCAUC, PRAUC and Brier scores) are calculated after 10 repetitions of the 10-fold cross-validation; for metrics based on Youden's index cut-off, the bootstrap method was used. PPV indicates positive predictive value; NPV, negative predictive value; AUC, area under curve; ROC, receiver-operating characteristic; PR, precision-recall.

	ML-Ours	Random Forests	Support Vector Machines	Logistic Regression	K-Nearest Neighbors
**Sensitivity**	0.72 (0.68-0.76)	0.63 (0.59-0.68)	0.60 (0.56-0.64)	0.61 (0.57-0.66)	0.51 (0.47-0.56)
**Specificity**	0.68 (0.64-0.71)	0.73 (0.69-0.77)	0.60 (0.56-0.64)	0.69 (0.65-0.73)	0.72 (0.68-0.75)
**PPV**	0.66 (0.61-0.69)	0.67 (0.62-0.71)	0.56 (0.51-0.60)	0.63 (0.58-0.67)	0.61 (0.56-0.65)
**NPV**	0.74 (0.71-0.78)	0.70 (0.66-0.74)	0.64 (0.60-0.68)	0.68 (0.64-0.72)	0.64 (0.60-0.67)
**F1 score**	0.69 (0.65-0.72)	0.65 (0.61-0.68)	0.58 (0.54-0.61)	0.62 (0.58-0.66)	0.56 (0.51-0.59)
**ROC AUC**	0.82 (0.47-1.00)	0.76 (0.35-1.00)	0.67 (0.18-0.93)	0.73 (0.24-0.98)	0.70 (0.19-0.96)
**PR AUC**	0.82 (0.50-1.00)	0.80 (0.42-1.00)	0.72 (0.36-0.94)	0.78 (0.39-0.98)	0.67 (0.37-0.93)
**Brier score**	0.19 (0.13-0.27)	0.21 (0.14-0.30)	0.24 (0.17-0.36)	0.22 (0.14-0.29)	0.22 (0.14-0.35)

Supplemental Table 4. Diagnostic performance of our approach and traditional ML models from repeated leave-one-out testing. Results are shown as median and 95% confidence intervals (CI). CIs for probability-based metrics (ROCAUC, PRAUC and Brier scores) are calculated using the set of measures obtained using out-of-fold predictions after 10 repetitions of the leave-one-out testing; for metrics based on discrete labels, the bootstrap method on accumulated predictions was used. ML indicates machine learning; PPV, positive predictive value; NPV, negative predictive value; ROC, receiver-operating characteristic; PR, precision-recall and AUC, area-under-curve.

	**ML-Ours**	**Random Forests**	**Support Vector Machines**	**Logistic Regression**	**K-Nearest Neighbors**
**Sensitivity**	0.66 (0.61-0.70)	0.49 (0.45-0.54)	0.65 (0.60-0.69)	0.61 (0.57-0.65)	0.52 (0.48-0.56)
**Specificity**	0.68 (0.65-0.72)	0.74 (0.70-0.77)	0.52 (0.48-0.56)	0.67 (0.63-0.70)	0.66 (0.62-0.69)
**PPV**	0.63 (0.58-0.66)	0.60 (0.55-0.64)	0.52 (0.48-0.56)	0.60 (0.56-0.64)	0.55 (0.51-0.59)
**NPV**	0.71 (0.68-0.75)	0.64 (0.61-0.68)	0.65 (0.60-0.69)	0.68 (0.64-0.72)	0.63 (0.59-0.67)
**F1 Score**	0.64 (0.61-0.67)	0.54 (0.50-0.58)	0.58 (0.54-0.61)	0.61 (0.57-0.64)	0.53 (0.50-0.57)
**ROCAUC**	0.73 (0.68-0.77)	0.69 (0.58-0.77)	0.59 (0.36-0.68)	0.68 (0.60-0.81)	0.62 (0.51-0.69)
**PRAUC**	0.60 (0.51-0.67)	0.65 (0.48-0.71)	0.54 (0.38-0.61)	0.61 (0.48-0.78)	0.51 (0.44-0.60)
**Brier**	0.22 (0.21-0.24)	0.22 (0.20-0.26)	0.25 (0.24-0.27)	0.23 (0.20-0.24)	0.24 (0.23-0.27)

Supplemental Figure 1. Precision-recall curves of the proposed approach and traditional ML models.

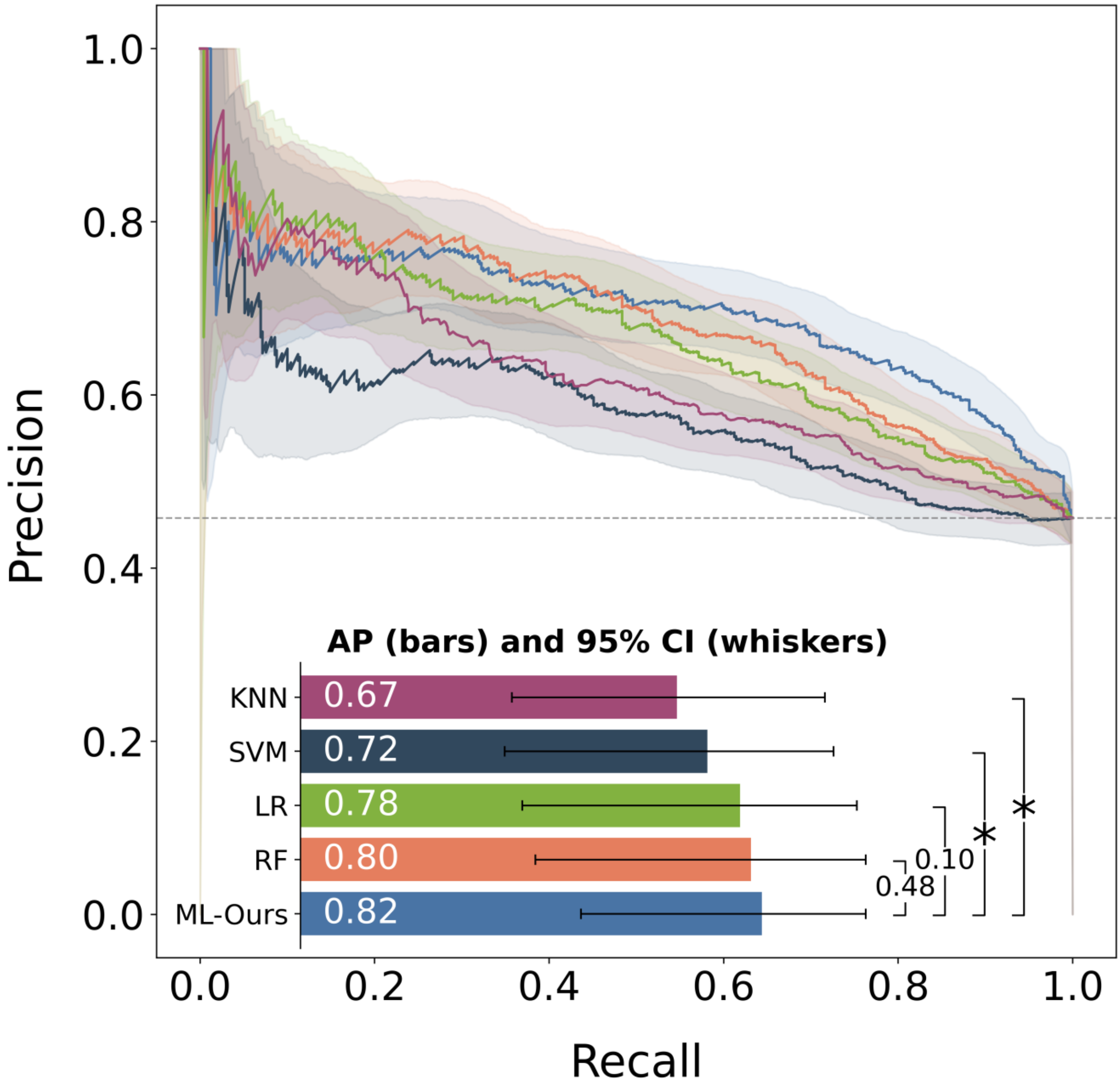

*P < .001 for AUC comparison by t-test

Supplemental Figure 2. Receiver-operating characteristic curves of the proposed approach and traditional ML models from leave-one-out testing.

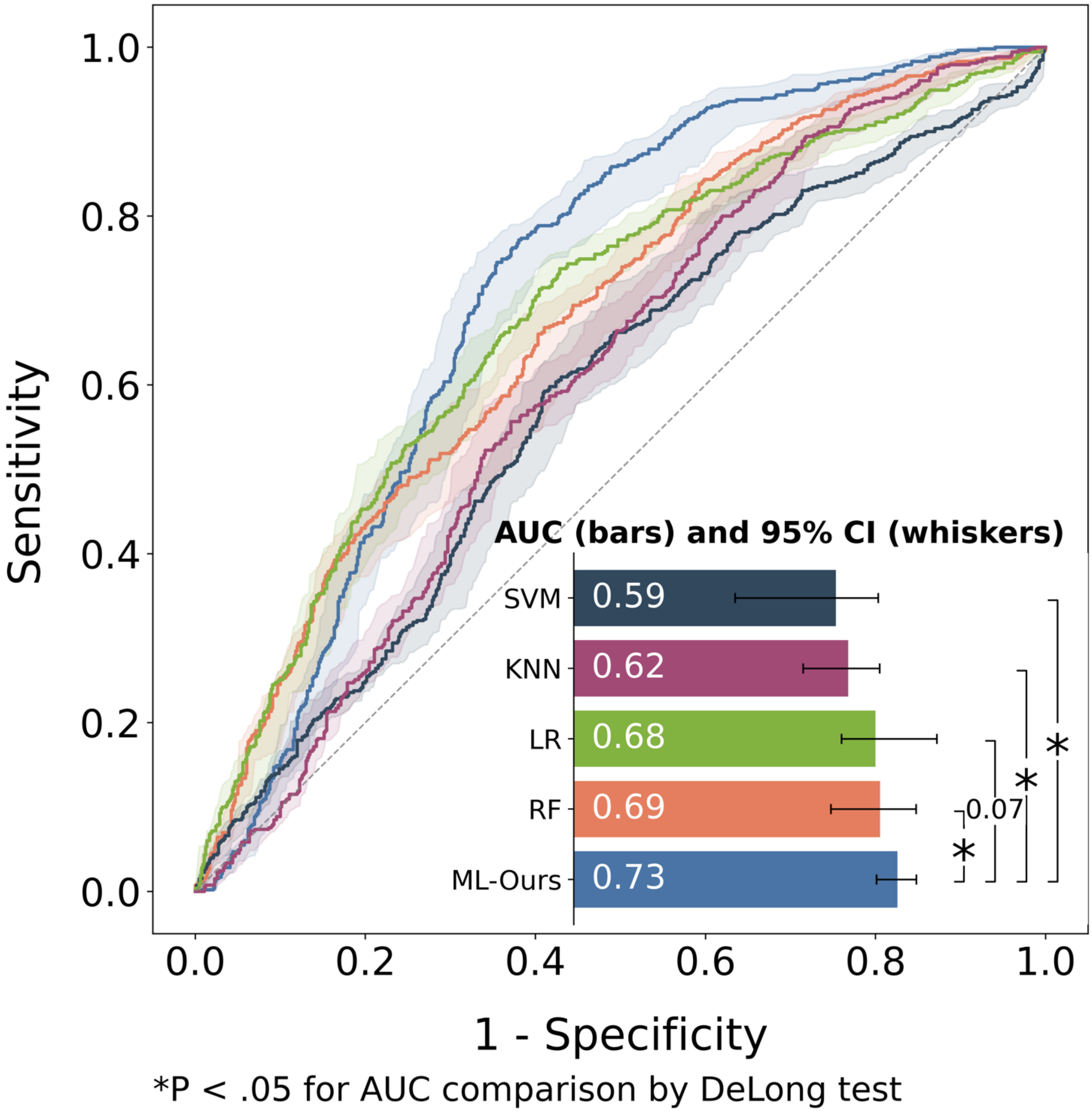

Supplemental Figure 3. Time required for the entire model building procedure by the proposed approach and traditional ML models.

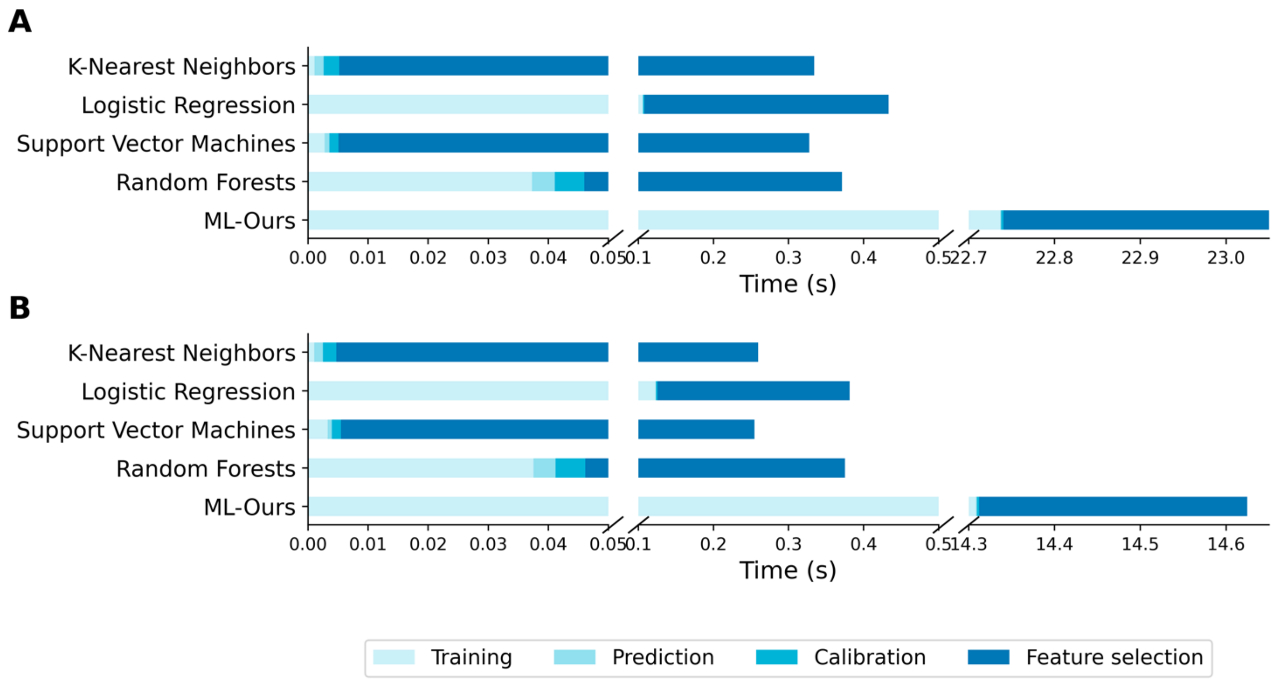

References

1. Hastie T, Tibshirani R, Friedman J. The Elements of Statistical Learning. Springer New York Inc.; 2001.
2. Lou Y, Caruana R, Gehrke J, Hooker G. Accurate intelligible models with pairwise interactions. *Proceedings of the 19th ACM SIGKDD international conference on Knowledge discovery and data mining*. Published online 2013. https://api.semanticscholar.org/CorpusID:11246170
3. Breiman L. Random Forests. *Mach Learn*. 2001;45(1):5-32. doi:10.1023/A:1010933404324
4. Hearst MA, Dumais ST, Osuna E, Platt J, Scholkopf B. Support vector machines. *IEEE Intelligent Systems and their Applications*. 1998;13(4):18-28. doi:10.1109/5254.708428
5. James G, Witten D, Hastie T, Tibshirani R. Statistical Learning BT - An Introduction to Statistical Learning: with Applications in R. In: James G, Witten D, Hastie T, Tibshirani R, eds. Springer US; 2021:15-57. doi:10.1007/978-1-0716-1418-1_2
6. Molinaro AM, Simon R, Pfeiffer RM. Prediction error estimation: a comparison of resampling methods. *Bioinformatics*. 2005;21(15):3301-3307. doi:10.1093/bioinformatics/bti499
